# Was der (Allgemein- und Viszeral‑)Chirurg über die Thromboseprophylaxe wissen sollte

**DOI:** 10.1007/s00104-021-01568-6

**Published:** 2022-02-11

**Authors:** Saskia Meißler, Rüdiger Braun-Dullaeus, Michael Hansen, Frank Meyer

**Affiliations:** 1 Klinik für Kardiologie und Angiologie, Universitätsklinikum Magdeburg A.ö.R., Magdeburg, Deutschland; 2 Klinik für Allgemein‑, Viszeral‑, Gefäß- und Transplantationschirurgie, Universitätsklinikum Magdeburg A.ö.R., Magdeburg, Deutschland

**Keywords:** Nierenerkrankung, Tumorerkrankung, Ambulante Operationen, Gerinnungserkrankung, Nutzen-Risiko-Bewertung, Kidney disease, Tumor disease, Outpatient surgery, Blood coagulation disorders, Benefit-risk assessment

## Abstract

Ein persistierendes Problem im klinisch-operativen Alltag ist die operationsassoziierte venöse Thrombembolie mit ihren möglichen Komplikationen. Zu den ureigenen und elementaren Aufgaben des Chirurgen, sowohl des Operateurs als auch des klinisch tätigen Mediziners, gehören daher die kompetente und verlässlich realisierte Thrombembolieprophylaxe. Zahlreiche präventive Ansätze wurden entwickelt und im täglichen Management etabliert, ein Restrisiko für eine Thromboseentwicklung bleibt bestehen. Unter diesem Aspekt wurde eine Recherche insbesondere zur wissenschaftlichen Literatur mit Übersichts- und Leitliniencharakter zum Thema Risikostratifizierung, Prophylaxeverfahren allgemein und bei spezifischen Indikationen durchgeführt.

**Zusatzmaterial online:**

Zusätzliche Informationen sind in der Onlineversion dieses Artikels (10.1007/s00104-021-01568-6) enthalten.

## Abstract (en)

A persisting problem in the clinical operative routine is surgery-associated venous thromboembolisms with the possible complications. The competent and reliably realized prophylaxis of thromboembolism is part of the original and elementary tasks of the surgeon, both the operator as well as the clinically active physician. Many preventive approaches were developed and established in the daily management but a residual risk for development of thrombosis still remains. Under this aspect a search was carried out particularly with respect to scientific literature with review and guideline character on the topic of risk stratification, prophylactic procedures in general and for specific indications.

Die operationsassoziierte venöse Thrombembolie mit ihren möglichen Komplikationen stellt ein persistierendes Problem im klinisch-operativen Alltag dar. Obwohl zahlreiche präventive Ansätze suffizient entwickelt, evidenzbasiert bestätigt und im täglichen Management etabliert wurden, bleibt ein patienten-, diagnose- und prozedurabhängiges Restrisiko für eine Thromboseentwicklung bestehen, deren kompetente und verlässlich realisierte Prophylaxe zu den ureigenen und elementaren Aufgaben des Chirurgen (wie auch jeglichen Operateurs und klinisch tätigen Mediziners), so auch in der Allgemein- und Viszeralchirurgie, gehören.

Das Ziel der narrativen kompakten Kurzübersicht ist es, basierend auf

i) ausgewählten und vor allem aktuellen Referenzen der medizinisch-wissenschaftlichen Literatur, insbesondere mit Übersichts- und Leitliniencharakter, und

ii) einschlägigen Erfahrungen aus dem eigenen klinischen Alltagsmanagement

Grundsätze der perioperativen Thromboseprophylaxe, insbesondere im allgemein- und viszeralchirurgischen Kontext unter besonderer Betonung aktueller Entwicklungen und weiterführender Trends, zu umreißen.

## Methode

Für die Recherche wurden aktuelle Leitlinien internationaler Fachgesellschaften zum Thema Thromboseprophylaxe in der Allgemeinchirurgie („general surgery“) sowie Publikationen im Zeitraum der letzten 10 Jahre der Datenbank Medline herangezogen. Weiterhin wurden Fachinformationen der in Deutschland zur Thromboseprophylaxe zugelassenen Medikamente berücksichtigt.

Allgemeine Aspekte

*Venöse Thrombembolien* (*VTE*) sind neben Blutungen eine gefürchtete Komplikation nach chirurgischen Eingriffen. Unter VTE wird das Auftreten *tiefer Venenthrombosen *(*TVT*) sowie *pulmonaler Thrombembolien *(*PE*) zusammengefasst. Thrombembolische Ereignisse sind, wie bereits 1856 von Rudolf Virchow als Virchowsche Trias beschrieben, durch drei entscheidende Einflussfaktoren bedingt:

- Stase,
- Hyperkoaguabilität (und)
- Endothelverletzung.

Im Rahmen eines chirurgischen Eingriffs können durch Immobilisation, Hypovolämie, Hypotonie, relative Stase, Verletzung von Geweben und Gefäßen alle drei Bedingungen gleichzeitig auftreten.

Noch in den 1980er- und 1990er-Jahren des letzten Jahrhunderts starben allein in den USA ca. 50.000 Menschen pro Jahr an den Folgen einer VTE nach einem operativen Eingriff, die postoperative Thromboserate lag bei bis zu 50 % [1].

Die VTE-Inzidenz der Normalbevölkerung beträgt ca. 0,1 % [2]. Ohne angemessene prophylaktische Maßnahmen kann diese bei allgemeinchirurgisch hospitalisierten Patienten 15–40 % betragen, konnte aber in den letzten Jahrzehnten durch die Etablierung von Leitlinien und Thromboseprophylaxeregimes auf 5–15 % gesenkt werden [2, 3].

Die 30-Tages-Mortalität postoperativer allgemeinchirurgischer Patienten, welche eine VTE erleiden, ist mit 11 % weiterhin als hoch zu bewerten [3]. Epidemiologisch erklärt sich dies u. a. durch die Zunahme von Faktoren wie Tumorerkrankungen, Alter und Immobilität, welche das Auftreten von Thrombosen begünstigen und die Mortalität erhöhen [4]. So liegt die VTE-Rate bei Tumorpatienten bis zu 7‑fach höher als bei gesunden Patienten, das Mortalitätsrisiko erhöht sich sogar um das 30-Fache [5]. Die Zahl der Krebsneuerkrankungen in Deutschland steigt stetig und aufgrund der demografischen Entwicklung wird zwischen 2015 und 2030 nochmals mit einer Zunahme von rund 23 % gerechnet [6]. Im Jahr 2017 war jeder zweite allgemeinchirurgische Patient in Deutschland älter als 65 Jahre [7]. Mit dem Alter kommen weitere Komorbiditäten und VTE-Risikofaktoren wie Immobilität hinzu.

Ein weiterer nicht zu vernachlässigender Einflussfaktor sind die immer präziser werdenden Bildgebungsverfahren, wodurch heute bereits kleinste Thrombosen und Embolien detektiert werden können, die zuvor verborgen blieben. Deren Erfassung hat jedoch klinische Relevanz, da auch für kleine, asymptomatische VTE-Ereignisse eine höhere Mortalitätsrate nachgewiesen werden konnte [3].

Das Auftreten venöser Thrombembolien zu verhindern, sollte daher weiterhin eine zentrale Rolle im modernen peri- und postoperativen Management einnehmen, um Komplikationen und schwere, potenziell tödliche Folgen abzuwenden. Zu den schweren Folgeerkrankungen eines TVT-Ereignisses gehören z. B.

- chronische Schmerzen,
- venöse Insuffizienz (und)
- Ausbildung eines postthrombotischen Syndroms (PTS) mit Ulzerationen.

Eine chronisch-venöse Insuffizienz mit Indikation für eine dauerhafte Kompressionstherapie bedeutet u. U. ein lebenslanges Tragen von Kompressionstrümpfen und stellt für viele Betroffene eine starke Beeinträchtigung der Lebensqualität dar. Allein das Anziehen ist gerade für ältere Patienten häufig nur mit Hilfe zu bewältigen.

Als Folge einer Lungenembolie kann neben den

- akuten lebensbedrohlichen Komplikationen wie Schock und Herz-Kreislauf-Versagen,
- in 3–4 % der Fälle langfristig eine chronisch-thrombembolische pulmonale Hypertonie (CTEPH)

resultieren. Unbehandelt führt diese rasch zu respiratorischer Insuffizienz und Tod aufgrund von Rechtsherzversagen, die 5‑Jahres-Überlebensrate liegt nur bei 50–60 % [8]. Ist ein VTE-Ereignis eingetreten, dann muss schnellstmöglich eine therapeutische Antikoagulation initiiert werden, was gerade postoperativ mit schweren Blutungskomplikationen assoziiert sein kann.

Letztendlich müssen auch ökonomische Aspekte Berücksichtigung finden, da bei Patienten, die peri- oder postoperativ eine VTE erleiden, mit 1,5-fach höheren Folgekosten für Therapien, Arztkonsultationen und Hospitalisierungen gerechnet werden muss [9].

Die übereinstimmende Empfehlung der aktuellen internationalen Leitlinien ist daher eine präoperative Evaluation des *individuellen* VTE-Risikos für *jeden* Patienten und die Festlegung eines Prophylaxeschemas unter Berücksichtigung des aktuellen Blutungsrisikos. Das Schema sollte nicht starr sein, sondern stetiger Reevaluation und ggf. Anpassung an den Krankheitsverlauf unterliegen. Dies ist u. a. die Lehre aus früheren fixen Prophylaxeschemata, mit denen zwar eine Reduktion der VTE-Rate von 50–80 % erreicht werden konnte, aber von denen der einzelne Patient aufgrund höherer Blutungsraten in der Summation nicht profitierte [10].

Weiterhin haben sich strukturelle Voraussetzungen wie klinikinterne SOPs (Standard Operating Procedure[s]) und regelmäßige Schulungen des gesamten pflegerischen und ärztlichen Personals als VTE-protektiv erwiesen [8].

Auch die poststationäre Situation muss beachtet werden. Aufgrund der zunehmend kürzeren Liegezeiten müssen gerade Patienten mit hohem VTE-Risiko in der Lage sein, für einen bestimmten Zeitraum Prophylaxemaßnahmen auch ambulant fortzusetzen oder es muss entsprechende Hilfe organisiert werden. Zum Entlassmanagement sollten daher neben der Patientenaufklärung über Basismaßnahmen die Anwendung prophylaktischer Maßnahmen und Symptome möglicher Komplikationen auch genaue Anweisungen für den Hausarzt und den Physiotherapeuten gehören.

Die aktuellen deutschen S3-Leitlinien zur Thromboseprophylaxe stammen bereits aus dem Jahr 2015. Die nachfolgende Arbeit soll daher einen kurzen Überblick über den aktuellen wissenschaftlichen Kenntnisstand geben und die Highlights der neuesten internationalen Empfehlungen zur perioperativen Thromboseprophylaxe allgemeinchirurgischer Patienten zusammenfassen [2, 11–15].

## VTE-Risikostratifizierung – Risiko-Nutzen-Einschätzung

Bei Patienten mit mittelgradigem und hohem VTE-Risiko wird eine medikamentöse Prophylaxe empfohlen. Damit der einzelne Patient jedoch profitiert, muss das individuelle VTE-Risiko das Blutungsrisiko übersteigen. Vor der Anwendung einer medikamentösen Prophylaxe sollte daher die genaue Risiko-Nutzen-Abwägung erfolgen und im Verlauf einer regelmäßigen Reevaluation unterliegen. Das individuelle Thromboserisiko eines Patienten setzt sich zusammen aus dem *dispositionellen* und dem *expositionellen* Risiko [2].

Unter *dispositionellem* Risiko versteht man die Summe der patienteneigenen vorbestehenden Triggerfaktoren, für die ein erhöhtes VTE-Risiko besteht. Je nach Stärke des VTE-Risikos unterscheidet man *Minor-, intermediäre *und* Major-Triggerfaktoren *(Tab. 1).

**Table 1:** 

*Dispositionelle Risikofaktoren für VTE*	
Major-Faktor (VTE-Risiko stark erhöht)	Alter > 75 Jahre
	Frühere VTE
	Thrombophilie/Gerinnungsstörung
	Aktive Tumorerkrankung
	Immobilisation (u. a. Apoplex mit Parese, Trauma mit Parese) < 1 Monat
Intermediärer Faktor (VTE-Risiko mittelgradig erhöht)	Adipositas (BMI > 30)
	Alter > 60 Jahre
	Thrombophilie/VTE bei Verwandtem 1. Grades
	Kreatinin-Clearance < 30 ml/min
	Orale Kontrazeptiva (1. und 3. Generation)
Minor-Faktor (VTE-Risiko leicht erhöht)	Schwangerschaft/post partum (< 1 Monat)
	BMI > 25
	Alter 41–60 Jahre
	Hospitalisierung mit akuter internistischer Erkrankung (Herz- oder respiratorische Insuffizienz)
	Entzündliche Darmerkrankung
*Expositionelle Risikofaktoren für VTE*	
Immobilisation (> 72 h)	
Lange Operationsdauer (> 45 min)	
Intubationsnarkose > 30 min	
Große intraabdominelle Operation	
Zentralvenöser Zugang (zentraler Venenkatheter)	

*BMI* Body-Mass-Index, *VTE* venöse Thrombembolie

Zu den Faktoren mit hohem Risiko gehören u. a. eine vorhergehende TVT, eine aktive oder in Therapie befindliche Tumorerkrankung, ein kürzlich erlittener Apoplex mit Paresen sowie angeborene Thrombophilien [16]. Bei Frauen ist eine hormonbedingte erhöhte Thromboseneigung zu beachten. Während Schwangerschaft und die Post-partum-Phase noch zu den Minor-Triggern gezählt werden, können Hormonpräparate je nach Zusammensetzung stark thrombogen wirken. So hat eine Hormontherapie mit Östrogenen eine deutlich höhere VTE-Rate als Kombinationspräparate mit Progesteron. Orale Kontrazeptiva der 1. und 3. Generation sind ebenfalls deutlich prothrombogener in ihrer Wirkung als Zweitgenerationspräparate [17]. Die britischen Leitlinien empfehlen die Umstellung oder das Absetzen östrogenhaltiger Präparate 4 Wochen vor einem Elektiveingriff [11]. Die aktuellen deutschen Leitlinien sprechen sich gegen ein Pausieren einer bestehenden kontrazeptiven Medikation aus, empfehlen aber eine medikamentöse und physikalische Thromboseprophylaxe [2]. Das perioperative Management sollte daher in Konsens mit den Kollegen der Gynäkologe erfolgen, um Komplikationen wie Blutungen zu vermeiden [18].

*Expositionelle* Risikofaktoren sind passager und durch den Eingriff bedingt (Tab. 1). Sie differieren je nach Art und Dauer des Eingriffs. Lange, ausgedehnte Operationen, eine prolongierte postoperative Immobilität, aber auch eine notfallmäßig durchgeführte Operation sowie offen-chirurgische abdominelle oder pelvine Eingriffe besonders im Rahmen einer Tumoroperation sind mit einem hohen VTE-Risiko vergesellschaftet. Eingriffe mit niedrigem VTE-Risiko sind u. a. die laparoskopische Cholezystektomie, Appendektomie und die inguinale Herniotomie [9, 14].

Meist bestehen mehrere VTE-Risikofaktoren. So kann die Kombination schwächerer oder expositioneller und dispositioneller Faktoren zu einem höheren individuellen VTE-Risiko führen.

Zur Risikostratifizierung wurden daher in der Vergangenheit zahlreiche Scoresysteme entwickelt. Allgemeinchirurgische Patienten werden dabei u. a. im Caprini-Score und im Roger-Score abgebildet [3, 16]. Die Anwendung ist jedoch nicht unstrittig, da die Scores nicht prospektiv validiert sind. Vor allem der Caprini-Score wird aber von vielen internationalen Leitlinien empfohlen [12, 14, 15]. Dieser nimmt eine Einteilung in fünf Risikogruppen (sehr gering, gering, moderat, hoch, höchstes VTE-Risiko) vor. In den meisten Leitlinien [11–15], so auch der aktuellen S3-Leitlinie [2], werden bei den Therapieempfehlungen aber nur drei Gruppen unterschieden mit einem niedrigen, mittelgradigen und hohen VTE-Risiko. Scoreberechnungen kosten Zeit, die letztendliche Einordnung und Therapieentscheidung erfolgt nach ärztlichem Ermessen. Die aktuelle deutsche S3-Leitlinie nimmt daher Abstand von einem bestimmten Scoresystem, das britische National Institute for Health and Care Excellence (NICE) entwickelte ein eigenes, in der täglichen Routine umsetzbareres System, das „NICE-department of health VTE risk assessment tool“ [19].

**Figure 1:**
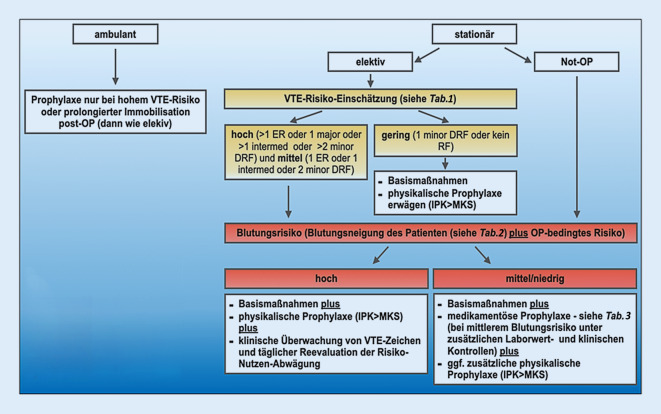

Im Rahmen dieser Arbeit wurde daher in Anlehnung an Caprini [16] und Sichtung der o. g. Leitlinien ein vereinfachtes praxisnahes Flussschema zur Risikostratifizierung in der Allgemeinchirurgie entwickelt (Abb. 1) und die wichtigsten Risikofaktoren für VTE-Ereignisse als Kurzübersicht (Tab. 1) zusammengefasst.

Vor Anwendung einer medikamentösen Prophylaxe muss neben dem VTE- auch das Blutungsrisiko evaluiert werden. Zahlreiche Faktoren mit erhöhter peri- oder postoperativer Blutungsgefahr sind bekannt. Auch hier spielen sowohl Komorbiditäten des Patienten, die eine erhöhte Blutungsneigung bedingen (Tab. 2), als auch eingriffsspezifische Faktoren zusammen. Validierte Scoresysteme für chirurgische Patienten fehlen jedoch [2]. Gerade bei schwerkranken, multimorbiden Patienten bleibt die Einschätzung des Blutungsrisikos somit eine erfahrungsbasierte Entscheidung des Operateurs. Bei angeborenen Hämophilien, gleichzeitig bestehenden internistischen oder neurologischen Erkrankungen, die ein hohes Blutungsrisiko bedingen, kann sich eine präoperative interdisziplinäre Konsultation als hilfreich erweisen.

**Table 2:** 

Blutungsrisiko	
Hoch	Intrakranielle Blutung
	Akuter Apoplex
	Schweres Trauma
	Kürzlich chirurgischer Eingriff am Zentralnervensystem
	Aktive Blutung
	Thrombozytopenie < 50 gpt/l
	Unkontrollierte arterielle Hypertonie
	Akute gastrointestinale Blutung
Mittel	Frühere Blutung
	Niereninsuffizienz
	Leberinsuffizienz
	Thrombozytopenie (< 100 gpt/l), funktionelle Thrombozytendefekte
	Hämophilie
	Aktive Ulzera
	Metastasierte Tumorerkrankung
	Alter > 80 Jahre
	Epidural‑/Spinalanästhesie vor < 4 oder innerhalb < 12 h geplant

Die verschiedenen Möglichkeiten der Prophylaxeverfahren sollen nun nachfolgend näher erläutert werden.

## Thromboseprophylaxeverfahren

Jeder Patient sollte im Rahmen eines operativen Eingriffs *Basismaßnahmen* zur Thromboseprophylaxe erhalten. Dazu gehören neben der

- Frühmobilisation,
- Bewegungsübungen und Physiotherapie auch eine ausreichende
- Hydratation.

Zusätzlich werden je nach individuellem VTE-Risiko eine *medikamentöse* und/oder *physikalische *Thromboseprophylaxe empfohlen [2].

### Physikalische Maßnahmen

Bei Patienten mit niedrigem VTE-Risiko oder Kontraindikation gegen eine medikamentöse Prophylaxe werden *physikalische Maßnahmen* eingesetzt. Hier haben sich

- medizinische Thromboseprophylaxestrümpfe (MTPS) und
- die intermittierende pneumatische Kompression (IPK)

bewährt.

Kompressionsstrümpfe führen zur Erhöhung der Flussgeschwindigkeit im tiefen Venensystem und wirken so der Entstehung von Thrombosen entgegen. Nach aktueller Datenlage wird der Nutzen von MTPS, abseits von entstauenden Maßnahmen, jedoch zunehmend infrage gestellt [20]. Man weiß heute, dass die alleinige MTPS-Anwendung gegenüber einer IPK oder der medikamentösen Prophylaxe keinen Vorteil bringt [21]. Außerdem ergibt sich kein Zusatznutzen einer MTPS-Therapie bei Kombination mit einer medikamentösen Prophylaxe [22, 23]. Deutsche und europäische Fachgesellschaften geben daher auch bei Patienten mit hohem VTE-Risiko nur noch eine „Kann“-Empfehlung für die Anwendung von MTPS zur Thromboseprophylaxe [2, 21]. Sollten MTPS angewendet werden, hat eine Kontrolle der Passform, eine Patientenschulung sowie die Überwachung von Komplikationen, wie beispielsweise Hautveränderungen, zu erfolgen [2].

Bei der IPK werden luftgefüllte Manschetten im Bereich der Beine angelegt. In definierten Intervallen und Druckniveaus erfolgt eine In- und Deflation von Luft. Die Muskeln werden komprimiert, das venöse Blut herzwärts gepumpt, was die Funktion der Muskelpumpen nachempfindet und der Stase in den Venen entgegenwirkt. Für die IPK konnte eine Nichtunterlegenheit gegenüber einer medikamentösen Prophylaxe und eine Überlegenheit gegenüber MTPS nachgewiesen werden [21]. Im Gegensatz zu den MTPS erbrachte die kombinierte Anwendung von IPK und medikamentöser Prophylaxe einen eindeutigen Zusatznutzen für Patienten mit hohem VTE-Risiko und wird von der ESA (European Society of Anaesthesiology) für diese Patienten empfohlen [21]. Jedoch ist eine nahezu kontinuierliche, auch nächtliche Anwendung der Kompressionssysteme notwendig [24]. Dies wird von den meisten Patienten als störend und schlafraubend empfunden und daher schlecht toleriert. Der limitierende Faktor ist somit die Compliance des Patienten [25]. In Deutschland konnte sich die IPK bisher nicht flächendeckend durchsetzen, auch wenn das Vorhalten einer IPK-Therapie für alle Kliniken empfohlen ist [2]. Sie bleibt hierzulande meist Domäne einzelner Fachdisziplinen wie der Neurochirurgie und Intensivmedizin. Des Weiteren ist eine intraoperative pneumatische Kompression in der bariatrischen Chirurgie empfohlen [26].

Bei beiden physikalischen Prophylaxeverfahren sind Kontraindikationen wie PAVK, Herzinsuffizienz, ausgeprägte Hypertonie, Entzündungen, Traumen und Neuropathien zu beachten. Eine frische tiefe oder oberflächige Beinvenenthrombose ist aufgrund einer erhöhten Emboliegefahr ebenfalls eine Kontraindikation für die IPK. Mögliche Kontraindikationen sollten vor der Verordnung stets evaluiert werden, um Komplikationen zu vermeiden.

Für Patienten mit hohem VTE-Risiko besteht prinzipiell eine weitere *physikalische Option *in Form einer

- „CAVA-Schirm“-Anlage.

Dabei handelt es sich um ein „Device“, was in die V. cava unterhalb der Nierenvenen eingebracht wird, um eine pulmonale Embolisation peripherer Thrombosen zu verhindern. Größere prospektive Studien fehlen, die bisher zur Verfügung stehenden Daten erbrachten aufgrund höherer Komplikationsraten keinen Patientenvorteil [27–29]. Der Einsatz des CAVA-Schirms ist daher heute weiterhin eine Einzelfallentscheidung, vorausgesetzt, dass bei hohem VTE- und Blutungsrisiko keine anderen Prophylaxemaßnahmen angewendet werden können. Auch international wird die CAVA-Schirm-Implantation nicht zur Anwendung im Rahmen einer VTE-Prophylaxe empfohlen [2, 11–15].

### Medikamentöse Thromboseprophylaxe

**Figure 2:**
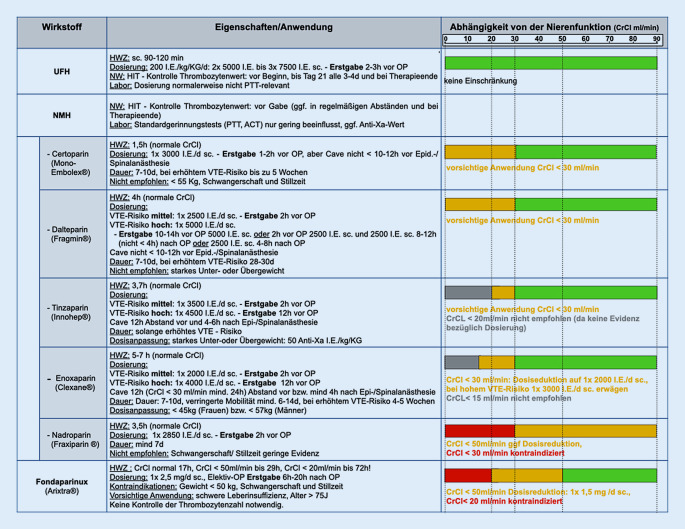

Bei Patienten mit mittelgradigem und hohem VTE-Risiko wird leitlinienübergreifend eine *medikamentöse Prophylaxe* empfohlen, so es das Blutungsrisiko zulässt ([2, 11–15]; Tab. 3). Der Beginn der medikamentösen Prophylaxe wird international unterschiedlich gehandhabt. Deutsche Fachgesellschaften empfehlen eine präoperative Prophylaxe ca. 12 h vor der Operation. Aber auch ein postoperativer Beginn in erhöhter Dosierung ist möglich [2]**. **Dabei sind je nach Eingriff und Narkoseform (z. B. Spinalanästhesie) spezifische, teils wirkstoffabhängige zeitliche Mindestabstände zum Eingriff zu beachten. Weiterhin hat eine Dosisanpassung an das VTE-Risiko und die Komorbiditäten (z. B. Niereninsuffizienz, Unter- oder Übergewicht) zu erfolgen (Abb. 2, Abschnitt „Medikamentöse Thromboseprophylaxe bei spezifischen Indikationen“). Die Prophylaxedauer bei mittlerem und hohem VTE-Risiko wird sowohl im ambulanten oder stationären Bereich in der Regel für mindestens 7 Tage empfohlen [2, 11]. Bei *hohem VTE-Risiko* (Malignomoperation, prolongierte Immobilisation) herrscht internationaler Konsens für eine prolongierte postoperative Thromboseprophylaxe von 4 bis 5 Wochen [2, 11–15].

**Table 3:** 

Empfehlungen zur medikamentösen Thromobseprophylaxe
*NMH >* *UFH*
*Fondaparinux in Spezialindikation* (abdomineller und bariatischer Eingriff, Kontraindikation gegen Heparin)
*Beginn:* Wenn möglich präoperativ/bei Aufnahme postoperativer Wiederbeginn nach Erreichen der Hämostase Narkosebesonderheiten (z. B. bei Spinalanästhesie) und Dosisanpassungen beachten
*Dauer:* mindestens bis zur Entlassung, in der Regel 3 bis 7 Tage bei hohem VTE-Risiko (z. B. Tumoroperation) bis zu 30 Tage

*NMH *niedermolekulare Heparine, *UFH* unfraktionierte Heparine, *VTE* venöse Thrombembolie

Zur medikamentösen Thromboseprophylaxe stehen

- niedermolekulare Heparine (NMH),
- unfraktionierte Heparine (UFH) und
- der synthetisch hergestellte Faktor-Xa-Inhibitor Fondaparinux

zur Verfügung.

Im Gegensatz zu orthopädischen Eingriffen (z. B. Hüfttotalendoprothese) gibt es aktuell keine Evidenz für den Einsatz direkter oraler Antikoagulanzien (DOACs) in der Allgemeinchirurgie [2, 12]. Einerseits fehlen prospektive Studien, andererseits kam es unter Prophylaxe mit DOACs bei internistischen Patienten zu signifikant erhöhten Blutungsereignissen [30]**.**

First-line-Empfehlung für allgemeinchirurgische Patienten der deutschen und europäischen Fachgesellschaften sind NMHs [2, 11, 12]. Sie besitzen ein gutes Wirkungs- und Risikoprofil.

So beobachtete man unter NMH im Vergleich zu UFH weniger relevante Blutungsereignisse bei größerer Reduktion von VTE-Ereignissen [31, 32]. Zudem haben sich NMH im Vergleich zu UFH auch als kosteneffektiver erwiesen [33]**. **Vor der Anwendung von NMH bei eingeschränkter Nierenfunktion sind Dosisanpassungen zu beachten, da eine überwiegend renale Elimination erfolgt (Abb. 2).

Eine weitere, allerdings häufiger unter UFH auftretende Komplikation ist die heparininduzierte Thrombozytopenie (HIT). Diese kann in Form der HIT II durch Bildung von Antikörpern gegen den Plättchenfaktor-4-Komplex zu lebensbedrohlichen arteriellen und venösen Thrombosen führen [34]. Um eine HIT frühestmöglich zu erkennen, wird die Kontrolle der Thrombozytenzahl für UFH bei Aufnahme, im Verlauf und nach Abschluss der Behandlung empfohlen [2]. Auch unter NMH wird eine Kontrolle empfohlen, bestimmte Zeitpunkte werden von den Leitlinien nicht gesetzt [2].

Kommt es unter oder nach Heparingabe zu einem VTE-Ereignis, muss eine HIT umgehend ausgeschlossen, die Heparintherapie sofort beendet und auf eine alternative Antikoagulation in therapeutischer Dosierung gewechselt werden. Der obligate HIT-Test darf nicht erst abgewartet werden [2, 12]. Zur Therapie einer akuten HIT sind in Deutschland nur Argatroban und Danaparoid zugelassen [2]. Fondaparinux darf zwar zur erneuten Prophylaxe nach HIT verwendet werden, aufgrund der fehlenden Datenlage aber nicht zur Therapie einer akuten HIT [34].

Fondaparinux zur Thromboseprophylaxe ist bei nichtorthopädischen Eingriffen weiterhin nur für spezielle Indikationen empfohlen wie eine bestehende Kontraindikation gegen Heparine und größere abdominelle und bariatrischen Eingriffe [2, 12, 34]. Auch hier sind zusätzlich Anwendungsbeschränkungen wie eine eingeschränkte Nierenfunktion und ein Mindestabstand von 24 h präoperativ und 6 h postoperativ zu beachten, um Blutungskomplikationen zu vermeiden [2, 34].

## Medikamentöse Thromboseprophylaxe bei spezifischen Indikationen

### Vor Notoperationen (ohne vorherige Antikoagulation)

Eine genaue Risikoevaluationen und präoperative Thromboseprophylaxe ist bei dieser Patientenklientel oft schwierig bzw. zeitlich nicht möglich. Die Datenlage ist sehr begrenzt. Allein die Schwere des Krankheitsbildes, die einen Noteingriff erfordert, bedingt ein erhöhtes VTE-Risiko [35]. Sollten keine absoluten Kontraindikationen wie große Blutungen vorliegen oder zu erwarten sein, wird aufgrund des erhöhten VTE-Risikos eine medikamentöse Thromboseprohylaxe in Form von Heparinen (NMH oder UFH) empfohlen [2]. Der Beginn der medikamentösen Prophylaxe sollte dabei möglichst sofort nach Aufnahme erfolgen, die übrigen Empfehlungen zur Thromboseprophylaxe unterscheiden sich nicht zum Elektiveingriff [36].

### Chronische kompensierte/dekompensierte Niereninsuffizienz

Bei Patienten mit schwerer Niereninsuffizienz (CrCl < 30 ml/min) besteht sowohl ein erhöhtes VTE-Risiko als auch Blutungsrisiko [37, 38]. So profitierten in der CERTIFY-Studie selbst Patienten mit schwerster Niereninsuffizienz von einer medikamentösen Thromboseprophylaxe [39]. Daher sollte gerade bei dieser Patientenklientel auf eine medikamentöse Thromboseprophylaxe geachtet werden, besonders wenn weitere VTE-Risikofaktoren hinzukommen. Niedermolekulare Heparine mit hohem Molekulargewicht sollten bei Abwesenheit von Kontraindikationen gegenüber UFH bevorzugt eingesetzt werden. Diese langkettigen NMHs mit großem Molekulargewicht und kurzer Halbwertzeit (HWZ) werden weniger renal eliminiert und erwiesen sich aufgrund geringerer Blutungsraten bei gleicher Wirksamkeit auch bei Patienten mit schwerer Niereninsuffizienz zunehmend sicherer als UFH [39].

Dazu gehören das Certoparin, Dalteparin und Tinzaparin. Certoparin und Dalteparin sind bei schwerer Niereninsuffizienz (CrCl < 30 ml/min) zwar mit Vorsicht anzuwenden, aber in prophylaktischer Dosierung nicht kontraindiziert [40, 41]. Auch der Einsatz von Tinzaparin ist bei schwerer Niereninsuffizienz prinzipiell nicht kontraindiziert, bei fehlender Evidenz bezüglich der Dosierung aber nicht empfohlen [42]. Enoxaparin ist bei schwerster Niereninsuffizienz (CrCl < 15 ml/min) nicht empfohlen [43]. Nadroparin und Fondaparinux sind kontraindiziert, da aufgrund des geringeren Molekulargewichts die renale Kumulationsgefahr und somit die Blutungsgefahr hoch sind ([27, 30, 44]; Abb. 2). Entscheidet man sich bei Patienten mit hohem Blutungsrisiko aufgrund eines hohen VTE-Risikos für die Gabe eines NMHs, wird empfohlen, diese mittels Faktor-Xa-Bestimmung zu überwachen [40–43].

Bei schwerster Niereninsuffizienz und Dialysepatienten ist eine medikamentöse Thromboseprophylaxe nur unter Vorsicht und nephrologischer Mitbetreuung anzuwenden.

Zur prophylaktischen Anwendung von NOACs gibt es aktuell keine Evidenz bei allgemeinchirurgischen Patienten mit Niereninsuffizienz [2].

### Tumorerkrankung als Haupt- oder Nebendiagnose

Das Risiko, im Krankheitsverlauf eine VTE zu erleiden, liegt für Tumorpatienten bei ca. 10 % [45]. Der Versuch, Tumorpatienten daher präventiv mit einer Thromboseprophylaxe zu versorgen, erbrachte aufgrund hoher Blutungskomplikationen keinen Überlebensvorteil [46]. Neben einem hohen VTE-Risiko besteht bei den meisten Patienten aufgrund von Gefäßerosionen, gerinnungswirksamen Therapien, Thrombozytopenien oder Metastasierung ein erhöhtes Blutungsrisiko. Demgegenüber haben bestimmte Tumorentitäten ein besonders hohes VTE-Risiko gezeigt. Dazu gehören unter anderem das Pankreaskarzinom, gastrointestinale Tumoren, Hirntumoren, Leukämien und das Ovarialkarzinom [47].

Tumorpatienten sollten daher möglichst *immer* eine perioperative medikamentöse Thrombosephrophylaxe erhalten, wenn es das Blutungsrisiko zulässt [2, 48]. Die beste Evidenz und das beste Sicherheitsprofil besteht für NMHs und Fondaparinux [2]. Die Prophylaxe sollte, wenn möglich, prolongiert, nach abdominellen und pelvinen Tumoroperationen auch bis zu 4 bis 5 Wochen erfolgen [2, 11–14]. Eine dauerhafte prophylaktische Dosierung unabhängig von einem Eingriff, einer Therapie oder nach stattgehabter nichttumorassoziierter VTE wird für Tumorpatienten weiterhin nicht empfohlen [2, 48].

### Tageschirurgische und ambulante Operationen

Ambulante Operationen bzw. die sog. Tageschirurgie beinhalten alle Eingriffe, bei denen der stationäre Aufenthalt maximal 24 h beträgt [49]. Die postoperative Therapie bzw. Versorgung des Patienten erfolgt ambulant. Prolongierte Immobilisation, mangelnde Volumensubstitution und Komplikationen wie Thrombembolien oder Blutungen entziehen sich klinischer Überwachungsmöglichkeiten.

Die ambulante postoperative VTE-Inzidenz liegt bei 0,04–0,15 % [50]. Jedoch konnten Subgruppen mit einem VTE-Risiko von bis zu 1,18 % identifiziert werden, die auch von einer medikamentösen Prophylaxe profitierten [50, 51]. Bei allen ambulanten Patienten sollte daher vor der Operation ein genaues „Assessment“ von VTE- und Blutungsrisiko erfolgen. Validierte Risikoscores für ambulante chirurgische Patienten fehlen bisher [51]. Retrospektive Analysen ergaben ähnliche VTE-Risikofaktoren wie für stationäre Patienten. Dazu gehören eine bestehende aktive Tumorerkrankung, Schwangerschaft, ein Alter > 60 Jahre, Body-Mass-Index > 35 kg/m^2^ und eine Operationsdauer > 120 min [50].

Die aktuelle ESA-Leitlinie befürwortet daher auch bei ambulanten Patienten die Verwendung des Caprini-Scores, bei der medikamentösen Prophylaxe werden NMH bevorzugt [49]. Die deutsche S3-Leitlinie empfiehlt bei der VTE-Risiko-Einschätzung wie bei stationären Patienten vorzugehen [2]. Patienten mit einem hohen VTE-Risiko und geringem Blutungsrisiko sollten eine medikamentöse Prophylaxe mit NMH oder UFH erhalten, wobei bei UFH das erhöhte HIT-Risiko und Kontrollen der Thrombozytenzahlen an Tag 5 und 14 zu beachten sind [2]. Dosierungen und Dauer unterscheiden sich in beiden Leitlinien nicht gegenüber Empfehlungen für stationäre Patienten [2, 49]. Bei hohem Blutungsrisiko ist auch bei ambulanten Patienten die medikamentöse Prophylaxe durch physikalische Maßnahmen zu ersetzen [2, 48, 50].

Weiterhin müssen die ambulanten Möglichkeiten des Patienten berücksichtigt werden, ggf. die medizinische Versorgung/Betreuung organisiert werden.

### Thrombozytenaggregationshemmung

Das Risiko einer Blutungskomplikation während eines chirurgischen Eingriffs wird durch Acetylsalicylsäure (ASS) um das 1,5-Fache erhöht. Dabei kommt es jedoch nicht zu einer Zunahme der Letalität [52]. Daher sollte ASS im Rahmen einer Sekundärprophylaxe – wenn möglich – auch perioperativ fortgesetzt werden, um vaskuläre Komplikationen zu vermeiden. Bei hohem VTE-Risiko ist zusätzlich die Anwendung einer medikamentösen Prophylaxe empfohlen. Das VTE-Risiko sollte aber das Blutungsrisiko übersteigen [53].

Beim Vorliegen einer dualen Thrombozytenaggregationhemmung (DAPT) ist präoperativ eine genaue Indikationsprüfung und Rücksprache mit der verordnenden Fachabteilung ratsam. So kann in einigen Fällen ein Thrombozytenaggregationshemmer pausiert oder in Abwägung mit dem perioperativen Blutungsrisiko vorzeitig beendet werden. Auch eine Umstellung von einem stark wirksamen ADP(Adenosindiphosphat)-Analogon wie Ticagrelor auf ein schwächer wirksames Thienopyridin wie Clopidogrel ist vereinzelt möglich. In anderen Fällen wie z. B. nach akutem Myokardinfarkt oder komplexen Stentimplantationen kann das Absetzen einer DAPT zu lebensbedrohlichen Komplikationen führen. Wenn möglich, sollte dann der chirurgische Eingriff bis nach Beendigung der DAPT-Therapie verschoben werden [53].

Muss ein chirurgischer Eingriff unter DAPT erfolgen, wird die Anwendung einer medikamentösen Thromboseprophylaxe aufgrund der erhöhten Blutungsgefahr auch bei hohem VTE-Risiko nicht empfohlen. Stattdessen sollte zusätzlich zu den Basismaßnahmen eine physikalische Prophylaxe durchgeführt werden [53].

### Prozedere bei bestehender Therapie mit Antikoagulanzien

Besteht präoperativ eine Indikation zur Antikoagulation, so unterliegt das perioperative Management gesonderten Leitlinien. In der Regel ist eine kurzzeitige Pausierung ohne parenterales Bridging möglich. Dies sollte aber individuell nach Abwägung von Thrombembolie- und Blutungsrisiko erfolgen. Es wird diesbezüglich auf die Leitlinien zum perioperativen Management bei therapeutischer Antikoagulation verwiesen [54, 55]. Diese Leitlinien müssen auch für reduzierte NOAK-Dosierungen, welche z. B. als reduzierte Erhaltungstherapie nach VTE-Ereignis (Rivaroxaban 1‑mal 10 mg/Tag oder Apixaban 2‑mal 2,5 mg/Tag) indiziert sein können, angewendet werden [56, 57].

Seit 2020 ist Rivaroxaban in niedriger Dosierung in Kombination mit ASS (Compass-Schema Rivaroxaban 2‑mal 2,5 mg/Tag und ASS 100 mg/Tag) für Patienten mit arterieller Polygefäßerkrankungen zugelassen, wodurch eine Reduktion kardiovaskulärer Ereignisse sowie der Mortalität nachgewiesen werden konnte [58]. Findet sich dieses Schema bei Aufnahme, sollte Rivaroxaban für den Eingriff pausiert werden und bei erhöhtem Thrombembolierisiko nach den üblichen Kriterien eine perioperative Thromboseprophylaxe zusätzlich zum ASS erfolgen (Abschnitt „Thrombozytenaggregationshemmung“).

### Gerinnungserkrankungen

Patienten mit Gerinnungsstörungen stellen eine besondere chirurgische Herausforderung dar. Sowohl eine

- erhöhte Gerinnungsneigung (*Thrombophilie*) als auch eine
- erhöhte Blutungsneigung (*Hämophilie*)

können unbeachtet zu massiven bis letalen Komplikationen führen, die durch ein korrektes perioperatives Management verhindert werden können.

Bereits eine genaue Patientenanamnese bei Aufnahme kann entscheidend sein. So sollten jedem Patienten Fragen nach früheren VTE-Ereignissen, Blutungen oder Komplikationen bei früheren Eingriffen gestellt werden [2]. Bei Frauen können Schwangerschaftskomplikationen und Aborte ein wichtiger Hinweis für eine Gerinnungsstörung sein. Zudem sollte die Familienanamnese erfasst werden. So haben 56 % der Patienten mit VTE-Ereignissen und 42 % mit VTE-Ereignissen bei Verwandten 1. Grades positive Thrombophiliemarker [59]. Ein generelles präoperatives Sceening auf Gerinnungsstörungen wird jedoch leitlinienübergreifend nicht empfohlen, kann aber bei Zustand nach VTE-Ereignissen oder positiver Familienanamnese erwogen werden [2, 60].

Häufige *Hämophilieformen* sind die Hämophilie A (Faktor-VIII-Mangel) und B (Faktor-IX-Mangel). Die häufigste angeborene Blutungsneigung ist das von-Willebrand-Syndrom. Dabei unterscheidet man genetisch 3 Typen, wobei Typ 1 aufgrund eines Mangels an von-Willebrand-Faktor (VWF) nur zu einer milden Blutungsneigung, Typ 3 aber bei Funktionsunfähigkeit des VWF zu schweren Blutungen führen kann. Das perioperative VTE-Risiko von Hämophiliepatienten ist als gering einzuschätzen, eine medikamentöse Prophylaxe wird daher regulär nicht empfohlen [2, 60]. Beobachtet und gefürchtet sind schwere Blutungskomplikationen. Als Hinweis für das Vorliegen einer möglichen Hämophilie sollte im Standardgerinnungslabor auf eine atypisch verlängerte aPTT („activated partial thromboplastin time“) geachtet werden [60].

Bei Vorliegen einer *Thrombophilie *kommt es je nach Ausprägung (homozygot, heterozygot) oder betroffenem Faktor zu einem leicht bis stark erhöhten VTE-Risiko [60]. Als milde Thrombophilie wird dabei die heterozygote Faktor-V-Leiden-Mutation und der Prothrombinpolymorphismus eingeordnet. Ein hohes VTE-Risiko, bis 15-fach erhöht im Vergleich zur Normalbevölkerung, stellen der Protein-C- und -S-Mangel sowie die homozygote Faktor-V-Leiden-Mutation (APC[aktiviertes Protein C]-Resistenz), der Antithrombinmangel und das Antiphospholipidsyndrom dar. Bei bekannter Thrombophilie ist daher immer eine medikamentöse Prophylaxe empfohlen, wenn nicht bereits eine Indikation zur therapeutischen Antikoagulation aufgrund einer schweren Thrombophilie besteht [60].

Bei bekannter oder beim Verdacht auf eine relevante Gerinnungsstörung sollte besonders vor elektiver Operation eine hämostaseologische Konsultation und Empfehlung zum perioperativen Management erfolgen [2, 60].

## Fazit

Aktuell gibt es zahlreiche Leitlinien verschiedener Fachgesellschaften, in denen sich teils unterschiedliche Empfehlungen finden (Tab. 4, siehe Zusatzmaterial online). Speziell für allgemeinchirurgische Patienten wird leitlinienübergreifend empfohlen: Patienten mit mittlerem oder hohem VTE-Risiko und niedrigem Blutungsrisiko sollten eine medikamentöse VTE-Prophylaxe erhalten, wobei NMH überwiegend vor UFH empfohlen werden. Für die Verwendung von NOACs gibt es bei allgemeinchirurgischen Patienten keine wissenschaftliche Evidenz.

Bei hohem Blutungsrisiko sind physikalische Maßnahmen indiziert, wobei die IPK effektiver als eine MTPS-Therapie ist. Eine alleinige oder zusätzliche MTPS-Therapie zur medikamentösen Prophylaxe wird zunehmend verlassen.

Wenn die Indikation für eine medikamentöse Prophylaxe gegeben ist, sollte die Erstgabe – wenn möglich – präoperativ erfolgen. Zeitabstände zur Operation und Dosierungen sind je nach Wirkstoff, individuellem VTE-Risiko, Komorbiditäten sowie der entsprechenden Narkoseform anzupassen. Prophylaktische Maßnahmen sollten in der Regel 7 Tage, bei hohem VTE-Risiko auch prolongiert werden. Die Arzt-Patienten-Interaktion in Form von genauer Anamneseerhebung und Patientenschulung stellt dabei einen entscheidenden Baustein zur Vermeidung peri- und postoperativer Komplikationen dar.

## Supplementary Information




