# Supplementary material for: What the (general and abdominal) surgeon should know about thrombosis prophylaxis
Source: Chirurgie (Heidelb). 2022 Feb 11;93(7):676–86. [Article in German] doi: 10.1007/s00104-021-01568-6 (PMC9246816; doi:10.1007/s00104-021-01568-6)
Supplement: Supplementary file 1 [file 104_2021_1568_MOESM1_ESM.docx]

**Tabellenlegenden**

**Tab. 1.** Dosierung und Anwendung der in Deutschland zur Thromboseprophylaxe in der Allgemeinchirurgie zugelassenen Wirkstoffe, Anpassung in

Abhängigkeit von der Nierenfunktion (ausgenommen Dialysepatienten) [30,40-44]


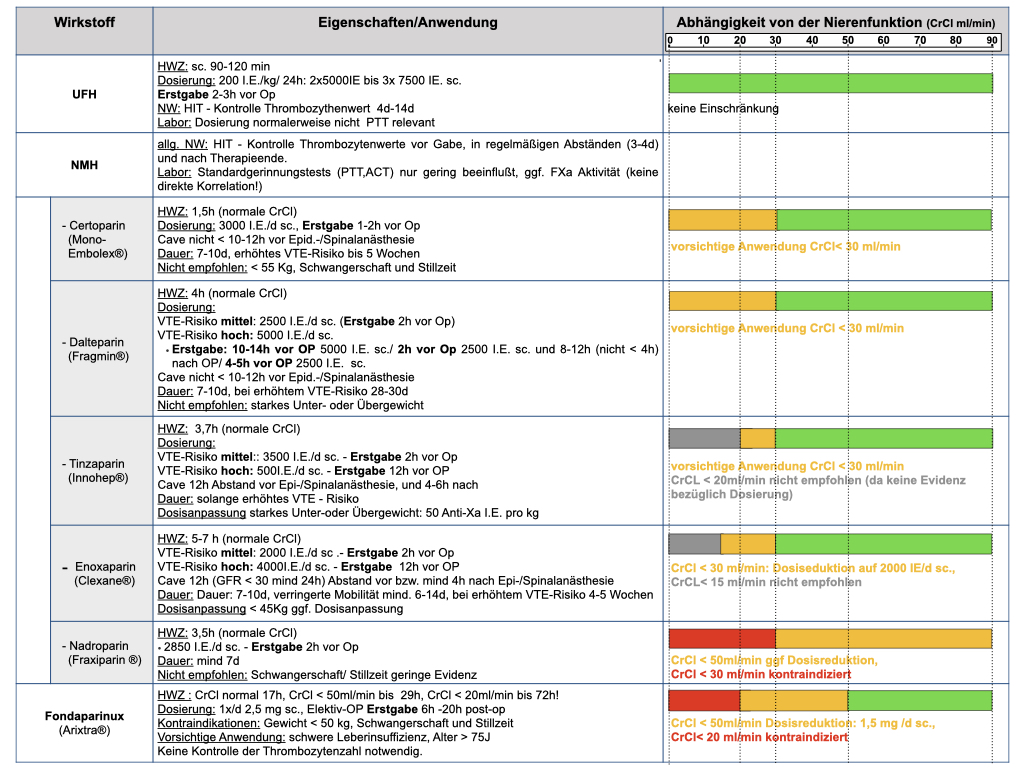


**Tab. 2.** Faktoren für ein erhöhtes peri- und postoperatives VTE-Risiko


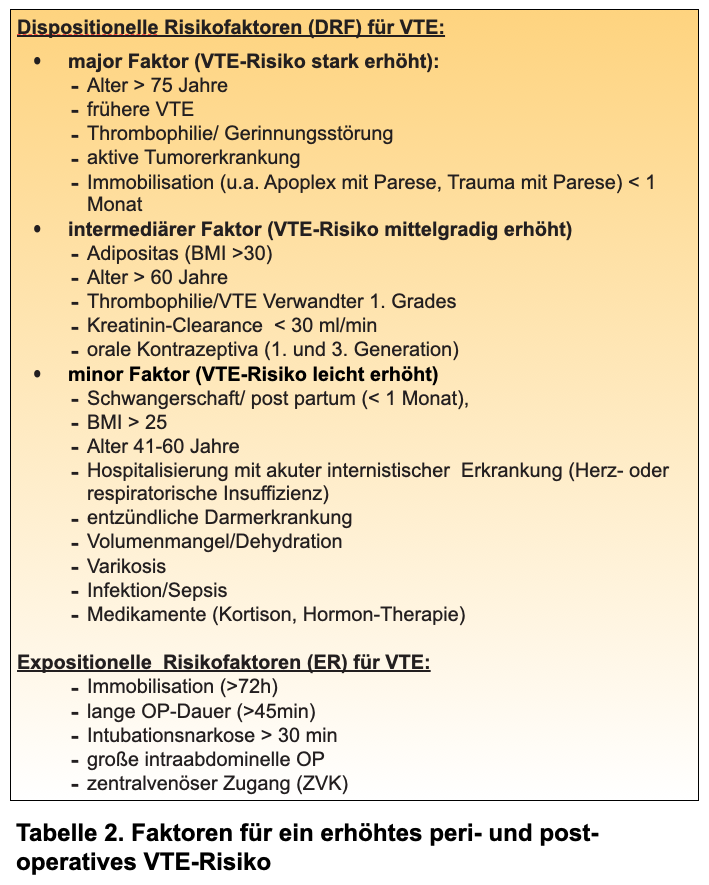


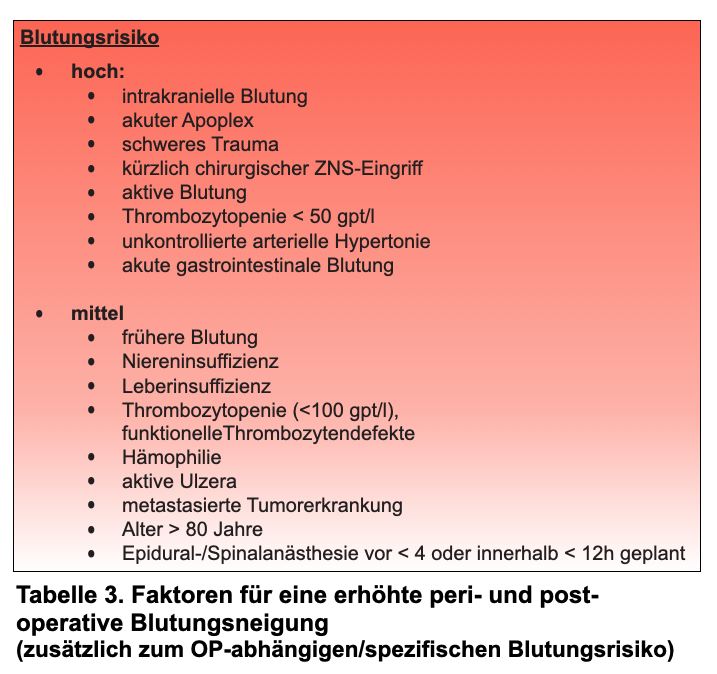
**Tab. 3.** Faktoren für eine erhöhte peri- und postoperative Blutungsneigung

**Tab. 4.** Kurzzusammenfassung der Empfehlungen nationaler und internationaler Leitlinien zur

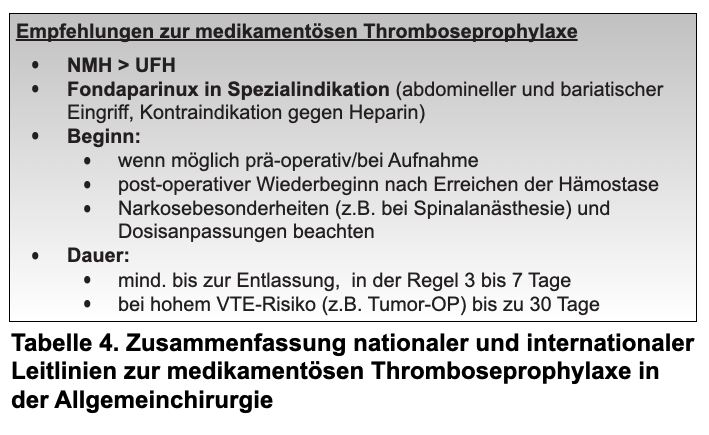
medikamentösen Thromboseprophylaxe bei allgemeinchirurgischen Patienten

**Tab. 5.** Zusammenfassung der Empfehlungen nationaler und internationaler Leitlinien bezüglich

der Thromboseprophylaxe bei allgemeinchirurgischen Patienten

***- bereitzustellen als QR-Code i.S. eines digitalen Supplements* (*online-Format*)**

| **Leitlinie** | **Empfehlung** |
| --- | --- |
| **Thrombosis** **Canada 2020**  [15] | Ermittlung VTE- (Caprini-Score) und Blutungsrisiko:   - niedriges VTE-Risiko: physikalisch erwägen - mittel-hohes VTE Risiko:   - Blutungsrisiko niedrig-mittel: NMH/UFH (Beginn: Hämostase, spätestens nach 12h, Dauer bis E, ggf. verlängert bis 30 d, +/- physikalisch   - Blutungsrisiko hoch: phys. Pr. (IPK > MKS), Reevaluation |
| American Society  of Hematology  **(ASH) 2019**  [13] | - große OP - medikamentös +/- physikalisch erwägen - hohes Blutungsrisiko: physikalisch - hohes VTE-Risiko: NMH/UFH + physikalisch - CCE laparoskopisch - keine Prophylaxe - hoch Risiko Tumor-OP - verlängerte Prophylaxe (bis zu 30 d) |
| National Institute  for Health and  Care Excellence  **(NICE) 2018** [11] | Ermittlung VTE- und Blutungsrisiko (eigener score: <http://www.nice.org.uk/guidance/ng89/resources>), gruppenspezifisch:   - Abdomin. Chir und bariatrisch:   - VTE>Blutungsrisiko: NMH oder Fondaparinux für mind 7 d, verlängert bis 28 d große Tumor-OP, MKS oder IPK (unter Beachtung KI)   - wenn Indikation zur med. Prophy. - Beginn schnellstmöglich und spätestens 14 h nach Aufnahme   - allgemeine Empfehlungen:   - - wenn möglich, sollten alle Östrogen-haltigen oralen Kontrazeptiva oder Hormone vor OP beendet werden, ggf. Alternative   - Antikoagulation - hohes VTE-Risiko - medikam. Prophylaxe während Unterbrechung erwägen.   - TAH: bei hohem VTE-Risiko ggf. zusätzlich medikamentös, sonst mech.   - Niereninsuffizienz: NMH/UFH, ggf. Dosisanpassung   - Tumorpatienten: NMH   ITS: NMH, phys., Reevaluation stetig (Risiko-Nutzen) |
|  |  |
| European Society of Anesthesiology (**ESA)** VTE Guidelines Task Force, *Eur J Anaesthesiol* TaJA) **2017**  [12,17,49,53,60] | Gruppenspezifisch, je nach VTE-Risiko zusätzlich zu Basismaßnahmen (BM):   - **medikamentöse Prophylaxe (MP):**   bariatische OP:   - VTE Risiko gering: IPK - VTE-Risiko mittel: 3.000-4.000 IE NMH s.c. alle 12 h - VTE-Risiko hoch (> 55 J, BMI > 55 kg/m^2^, früherer VTE, CVI/Varikosis, OSAS, Thrombophilie, pulmonale Hypertonie): 4.000-6.000 IE NMH s.c. alle 12 h, verlängert 10-15 d nach E - NMH > UFH   ambulant/“fast track“:   - VTE-Risiko nach Caprini-Score: - geringes OP-Risiko, VTE -Risiko gering: nur BM - geringes OP-Risiko + VTE-RF: NMH>UFH, bei zusätzlich hohem Blutungsrisiko: IPK - hohes-OP-Risiko: NMH>UFH plus IPK, bei zusätzlich hohem Blutungsrisiko: IPK - medikamentöse Prophylaxe 7 d > 3 d, Hoch-Risiko-Prozeduren bis 4 Wochen - Gabe: NMH 12 h vor OP, 6-8 h post-OP   ITS:   - immer NMH/UFH plus phys. (IPK) empfohlen (Ausnahme: hohes Blutungsrisiko oder Thr. < 50.000 - nur IK) - Niereninsuffizienz: UFH, Dalteparin oder reduzierte Dosis Enoxiparin, ggf. Messung der Anti-Xa-Aktivität - Leberinsuffizinez: Abwägung Blutungsrisiko, ggf. NMH, UFH - kein CAVA-Filter zur Prophylaxe empfohlen   HIT: 1. Wahl Argatroban  Pat. mit TAH   - ASS als alleinige Prophylaxe in der Allgemeinchir. nicht empfohlen - TAH plus Thromboseprophylaxe in der geringsten Dosierung bei mittlerem oder hohen VTE-Risiko - DAPT-Beginn vor med. Prophylaxe - bei erhöhtem Blutungsrisiko - IPK - Spinalanästesie: TAH weiter, medikamentöse Prophylaxe erst post-OP - TAH post-Op bei Hämostase, bei DAPT wenn keine KI Clopidogrel 24-48 h post-Op - auf Blutungszeichen achten - NSAR vermeiden   Thrombophilie- oder Hämophilie-Patienten: perioperatives Management nach hämostaseologischer Empfehlung, engmaschige klinische Kontrollen, ggf. Gerinnungsfaktorbestimmung  generelles Thrombophilie-Sceening prä-OP nicht empfohlen   - **phys. Prophylaxe (PP)**: IKP > MKS, nur bei hohem VTE-Risiko zusätzlich zu MP (PP allein bei hohem Blutungsrisiko)   CAVA Schirm nicht empfohlen |
|  |  |
| **AWMF S3 Leitlinie 2015**  [2] | BM,  D-Dimere nicht zur Risikostratifizierung  drei Risikogruppen: niedrig, mittel, hoch:  - VTE-Risiko niedrig: BM +/- physikalisch  - VTE-Risiko mittel-/hochgradig: BM + medik. Prophylaxe (NMH > UFH),  +/-phys.  physikalisch: MTPS od. IPK  Dauer: solange Risikofaktoren fortbestehen  Cava-Schirm nicht empfohlen  ASS nur in Einzelfällen  Bauch- und Beckeneingriffe:   - - NMH, alternativ Fondaparinux „kann“ Empfehlung   - Minimal/Laparoskopisch VTE-Prophylaxe wie bei offen chir. OP   - Dauer: 7 d; unabhängig, ob stationär oder ambulant, fortdauerndes VTE-Risiko - prolongiert, onkologische Eingriffe für 4 Wochen   Intensivpatienten:  medik. VTE-Prophylaxe, NMH > UFH, Hochrisikodosierung, bei KI: IPK > MTPS  Blutungsneigung, Niereninsuffizienz: low-dose UFH i.v.  Dauer - je nach RF (Erkrankung, Mobilität)  ambulant: gleiche Kriterien wie stationär, nach Entlassung Fortführung der Prophylaxe |
| USA: American  College of Chest  Physicians  **ACCP 2012**  [14] | Ermittlung VTE-Risiko (Caprini-, Roger-Score):  - sehr niedriges Risiko (< 0,5 %): keine Prophylaxe  - niedriges VTE-Risiko (ca. 1,5 %): physikalisch erwägen (IPK)  - mittleres VTE-Risiko (ca. 3 %): NMH/UFH oder mech. (IPK)  - hohes VTE-Risiko (ca. 6 %): NMH/UFH, +/- physikalisch (IKP/MTKS)  - höchstes VTE-Riskiko: NHM/UFH + physikalisch (IKP/MTKS)  - Blutungsrisiko hoch und VTE-Risiko hoch: phys. Pr. (IPK > MTKS),  Reevaluation  - hohes Risiko und abdominelle Tumor-OP - prolongierte Prophylaxe (4 Wo)  - für alle VTE-Risikogruppen: Prophylaxe mit CAVA-Filter oder KUS -  Überwachung nicht empfohlen.  KI Heparine, kein erhöhtes Blutungsrisiko - ASS oder Fondaparinux |
